# The association between physical exercise behavior and psychological resilience of teenagers: an examination of the chain mediating effect

**DOI:** 10.1038/s41598-024-60038-1

**Published:** 2024-04-23

**Authors:** Na Li, Dianguo Wang, Xiang Zhao, Zhen Li, Ling Zhang

**Affiliations:** 1 School of Physical Education, Shandong University of Science and Technology, Qingdao, China; 2 School of Physical Education, Huaibei Normal University, Huaibei, China

**Keywords:** Teenagers, Physical exercise behavior, Psychological resilience, Social sensitivity, Need to belong, Psychology, Health care

## Abstract

The health of young people is crucial for the future and development of a nation. It is the collective responsibility and imperative mission of society to ensure the holistic well-being, both physically and mentally, of young individuals. Therefore, it is essential to thoroughly comprehend the factors that influence their health in order to expedite the exploration of effective solutions. The objective of this study is to comprehend the mechanisms that underlie the correlation between physical exercise behavior and psychological resilience among teenagers, while also examining the mediating role played by social sensitivity and need to belong. So put forward the hypothesis: (1) physical exercise behavior can positively predict the psychological resilience. (2) Social sensitivity and need to belong plays a mediating role between physical exercise behavior and psychological resilience. (3) Social sensitivity and need to belong plays a chain mediating role between physical exercise behavior and psychological resilience. Using the cluster sampling method, a total of 1106 students (with an average age of 15.7 and a standard deviation of 0.598) who met the requirements were surveyed from Shandong Province in China. Standard scales were utilized to assess Physical Exercise Behavior, Psychological Resilience, Social Sensitivity, and Need to Belong. For data analysis, Pearson's correlation analysis and bias-corrected percentile Bootstrap method were sequentially conducted. (1) The present study did not find any significant methodological bias, and the observed correlations between physical exercise behavior, psychological resilience, social sensitivity, and need to belong were all statistically significant; (2) Based on the self-determination theory, this study elucidates the relationship between physical exercise behavior and psychological resilience among teenagers. The findings indicate that physical exercise behavior positively predicts the need to belong and psychological resilience, while negatively predicting social sensitivity. Similarly, social sensitivity negatively predicts the need to belong and psychological resilience. Moreover, the need to belong directly and positively predicts psychological resilience. Importantly, all hypotheses proposed in this paper were empirically supported. (3) The indirect effect of the path mediated by social sensitivity is 0.009, while the indirect effect of the path mediated by need to belong is 0.033. Additionally, the combined indirect effect of both social sensitivity and need to belong as mediating variables is 0.014. (4) The cumulative sum of all these indirect effects amounts to 0.056. Based on the self-determination theory, we propose a chain mediation model, specially, physical exercise behavior can significantly positively predict psychological resilience, among which, social sensitivity and need to belong play a significant mediating role between Physical exercise behavior and psychological resilience. In addition, the adoption of good physical exercise behavior can enhance the psychological resilience of adolescents by diminishing social sensitivity and augmenting the need to belong.

## Introduction

The stage of youth represents a pivotal period in life, serving as a catalyst for the overall development of individuals. Therefore, the cultivation and guidance towards teenagers' healthy growth are crucial factors that require meticulous attention. Simultaneously, this period presents a pivotal opportunity for the development of sound psychological needs, necessitating the acquisition of skills to effectively balance an array of burgeoning requirements^1^. However, due to the imbalance between the physiological and psychological development of teenagers, it is necessary to actively guide them, make them firm in their ideals and beliefs, and promote the shaping of healthy and ideal personality, so that they can live up to the historical responsibility entrusted by the times.

Teenagers' physical exercise behavior refers to the deliberate utilization of physical exercise methods to promote the holistic development of both physical and mental well-being. Good physical exercise behavior not only guides adolescents in establishing a healthy lifestyle but also serves as a preventive measure against psychological issues during their growth^2^. Psychological resilience primarily refers to the active response of individuals when confronted with external pressures and crisis situations, utilizing their inherent characteristics, abilities, social resources, and other protective factors to attain favorable adaptation outcomes in the face of adversity^3^. It encompasses the psychological adjustment exhibited by individuals while encountering or undergoing hardships, which stimulates their internal cognition, capabilities, or psychological traits^4^ and facilitates goal-oriented progress through proactive repair and optimization using both internal and external resources^5,6^. It has been proved that physical exercise can promote teenagers' mental health, such as improving self-efficacy and psychological resilience^7,8^. Also, Physical exercise can not only enhance physical fitness, but also promote mental health and improve sleep quality. Furthermore, it plays a crucial role in cultivating robust physical fitness and fostering a positive mindset among adolescents, thereby contributing to their overall physical and mental well-being^9^. Engaging in physical activity is imperative for developing a healthy and active lifestyle, as well as instilling lifelong health awareness and behaviors. Additionally, Physical exercise not only improves physical fitness but also enhances mental health and promotes better sleep quality^10^. Participation in physical exercise can promote the psychological development of teenagers in a positive direction^11^. Research shows that low physical activity actors are more likely to have unhealthy lifestyles and psychological problems due to less physical participation^12^. At the same time, research in the field of rehabilitation medicine and sports science points out that physical exercise has a positive effect on depression^13,14^. Therefore, teenagers' physical exercise behavior is closely related to their psychological resilience. In terms of coping with psychological problems, compared with medical treatment, psychological counseling and education, sports therapy based on physical exercise is the most green, economic and lasting way. The positive emotional experience generated in the process of sports and the resulting sense of happiness will weaken the psychological troubles and fatigue of teenagers in learning and life, and have a more significant role in improving positive emotions^15^.

## Social sensitivity, physical exercise behavior and psychological resilience

Teenagers are between childhood and adulthood, and they are highly sensitive, showing shyness and withdrawal in unfamiliar social situations^16^. Teenagers' social anxiety gradually emerged at the age of 13–19, mainly manifested as fear of social interaction, avoidance of social interaction, and some even appeared autonomic nervous disorder, so their social interaction ability declined^17^. Social sensitivity refers to the extent to which individuals perceive and are concerned about how their own behavior and social status are evaluated by others^18^. It encompasses the persistent fear, anxiety, or avoidance exhibited by adolescents towards unfamiliar environments or strangers during social interactions. Within these interactions, they demonstrate a heightened awareness of others' opinions and evaluations, leading them to adapt their behavior accordingly^19^. Moreover, social sensitivity entails individuals' ability to recognize, perceive, and comprehend subtle cues and contextual information within social exchanges. It also encompasses their capacity to empathize with others' emotions and ideas while possessing a deep understanding of societal norms and common conventions. Teenagers with strong social sensitivity are eager for others' recognition and pay more attention to social evaluation. When they are in a social situation, they will increase a strong sense of tension^18,20^, which may lead to fear and worry about the evaluation results, and experience the pressure of social interaction, resulting in more maladjustment problems^21,22^. Therefore, teenagers with heightened social sensitivity often experience discomfort in social situations and tend to harbor pessimistic expectations. They feel that others hold a negative evaluation of themselves, so they will have nervous and distressed emotional experiences, and thus have social avoidance behaviors to escape places where they perceive negative evaluation^23^. Faced with social difficulties and pressures, teenagers with high social sensitivity are under great psychological pressure, and they can not use internal and external resources to obtain good psychological adaptation, so their psychological resilience is reduced. If they cannot be controlled in time, they are more likely to form social avoidance and even social phobia. However, physical exercise behavior may negatively predict social sensitivity, and the positive emotional experience generated by physical exercise can improve teenagers' psychological problems and promote the development in a benign direction^24^. The engagement in sports activities concurrently exerts a substantial impact on enhancing the social skills, fostering solidarity and cooperation among adolescents, while simultaneously mitigating their social sensitivity. The weak social sensitivity is positively correlated with mental health, which will positively predict the psychological resilience of teenagers. In summary, physical exercise behavior may enhance teenagers' psychological resilience by negatively predicting their level of social sensitivity.

## Need to belong, physical exercise behavior and psychological resilience

Need to belong refer to a fundamental psychological and social requirement of individuals. It is widely recognized as a primary human motivation, playing a crucial role in the development and well-being of individuals^25^. In communication, we attribute oneself to a certain group and, as a member of the group, we perceive the emotional experience of being accepted, respected and supported^26,27^, including the psychological experience of cognition and emotion. The theory of need to belong believes that, as a social attribute, it is a basic human need to establish contact with others, so it is necessary to maintain the lowest sense of interpersonal belonging. The essence of need to belong is the psychological need to establish a harmonious interpersonal relationship with others and be accepted by groups^28^, therefor, in the presence of a deficiency in interpersonal belonging within a particular domain, individuals are encouraged to allocate their time and effort towards other collectives in order to establish a sense of belonging. While in sports, they can gain a strong sense of collective identity and organizational attachment, Higher emotional commitment will further improve sports experience and enhance the sense of belonging of teenagers^29^. Therefore, the inherent characteristics of sports projects enhance the sense of belonging and foster positive emotions associated with belonging, thereby potentially predicting psychological resilience in teenagers. Through engaging in physical exercise, teenagers develop a stronger sense of belonging and strive to fit in with their peers in a more positive manner, resulting in increased psychological resilience.

In addition, The desire for group acceptance, the avoidance of social exclusion, and the need for belonging have been identified as underlying factors contributing to individual social sensitivity in accordance with empirical studies^30,31^, teenagers with high social sensitivity are motivated to seek recognition from groups and organizations, as well as a sense of belonging and validation within the group. Consequently, they invest considerable effort in nurturing interpersonal relationships^32^. The actions of socially sensitive individuals are influenced by the perspectives and evaluations of other group members, which in turn facilitates the establishment and maintenance of positive personal and group relationships to fulfill their need for belonging. Conversely, teenagers with lower social sensitivity are more inclined to experience a satisfactory sense of group belonging^33^. It is evident that there exists a positive and complementary relationship between social sensitivity and the need to belong. Simultaneously, engagement in physical exercise behavior exhibits a positive correlation with psychological resilience and need to belong^7,8,34^, while displaying a negative association with social sensitivity^24^. Physical exercise behaviors have the potential to influence psychological resilience by mediating social sensitivity and need to belong. In summary, young individuals' engagement in physical exercise impacts social sensitivity and need to belong, thereby shaping robust psychological resilience.

The study is supported by self-determination theory, which describes the influence of motivation on behavior, and when any of these needs are blocked, it may have a negative impact on mental health^35^. The potential for self-determination can guide individuals to engage in behaviors that align with their interests and contribute to their personal development, thereby constituting the intrinsic motivation driving human behavior. When basic psychological needs are met, individuals are motivated to engage in certain behaviors, which in turn influence their actions, emotions, and cognition. According to self-determination theory, participating in physical exercise behaviors can satisfy three types of needs for individuals, including psychological, emotional, and competence needs. This has implications for their behaviors and emotions, including maintaining lower social sensitivity, enhancing the need to belong, and improving psychological resilience, thereby promoting the development of mental health.

**Figure 1:**
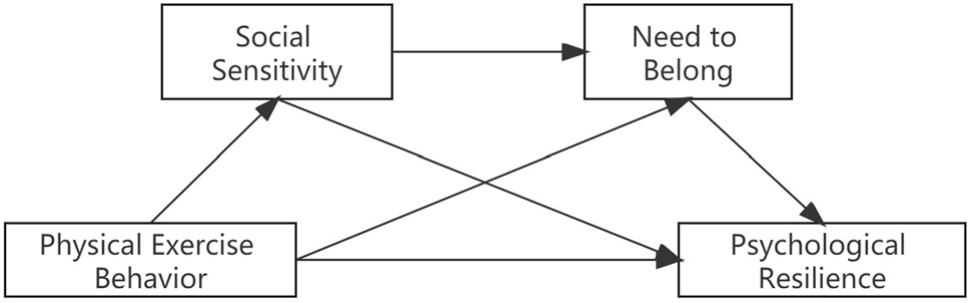
Conceptual model.

Existing studies at home and abroad have yielded relatively abundant findings, primarily focusing on the psychological resilience of adolescents and the impact of physical exercise on life satisfaction and social adaptation. However, these studies lack in-depth analysis of the underlying mechanisms, making it challenging to effectively address the physical and mental health issues faced by adolescents. In comparison to previous research, this study adopts a self-determination theory framework to explore the mechanism through which physical exercise behavior influences psychological resilience. By understanding the effects of physical exercise on adolescents' psychological well-being and adaptive abilities, this study provides valuable insights for investigating their physical health. In practical terms, our findings suggest that support should be provided in family education and school settings to actively attend to adolescents' psychological needs while enhancing their physical health. Therefore, this study aims to investigate the association between physical exercise behavior and psychological resilience among adolescents, while examining the following hypotheses: H1: Physical exercise behavior significantly predicts adolescent psychological resilience in a positive manner; H2: Social sensitivity and need to belong serve as distinct mediators between physical exercise behavior and psychological resilience; H3: Social sensitivity and need to belong act as sequential mediators between physical exercise behavior and psychological resilience (Fig. 1).

## Materials and methods

### Participants and procedure

Utilizing the cluster sampling technique, one public school was systematically chosen from each of the eight regions encompassing the eastern, central, and western areas of Shandong Province in China. The subsequent selection process involved the random sampling of three classes from each freshman cohort in every school, with each class consisting of 50 students. In total, 1200 questionnaires were distributed. All participants reported sharing the same ethnic background and socio-economic status. The students in the selected school have a PE class 3 times a week. The schools have enough places for exercise and fitness, and all the students are in good health. After removing the invalid questionnaires due to regular answers, missing data and other reasons, 1106 valid questionnaires were finally obtained, the recovery rate was 92.2%. In the valid questionnaire, 433 girls (39.2%) and 673 boys (60.8%) were selected, with an average age of 15.7 ± 0.59. The differential test conducted on the data revealed no statistically significant disparity among schools, Therefore, this study will analyze the data in its entirety.

Study consent was obtained from the school leader, the head teacher and the subject himself before testing. The questionnaire follows the principles of voluntary filling, data confidentiality and anonymous filling, and is tested by the collective test method. The questionnaire included control variables including participant age and gender, and the data were collected from 3 September to 1 November, 2022, and the questionnaire was completed within 30 min.

### Measures

#### The physical activity level scale (PARS-3)

The Physical Activity Level Scale was assessed by Deqing^36^, as a tool to measure teenagers’ participate in physical activities, this scale has been widely used in the study of physical activity participation. The scale contains three test questions, which mainly assess the amount of exercise in terms of exercise intensity, exercise time and exercise frequency. Each question was recorded as 1–5 points according to the grade. The mean score of the exercise amount was used as a quantitative indicator of the participants' exercise behavior evaluation. The average score of the exercise amount was used as a quantitative indicator of the participants' Physical exercise behavior evaluation. The internal consistency coefficient α = 0.84, and the half-degree of reliability is 0.82. In this study, the Cronbach's α coefficient of the scale was 0.82, while the Omega coefficient demonstrated a robust value of 0.79, indicating a high level of reliability.

#### Social sensitivity

The Social Sensitivity was evaluated by “The Social Sensitivity scale”, which was developed by Chen et al.^20^. The scale is used to measure teenagers’ social perception and attitudes. The scale included 13 items, with a grade 5 rating, 1 as "complete inconformity" and 5 as "full conformity". A higher score indicates a higher level of social sensitivity. The Cronbach's α coefficient of this scale was 0.88. In this study, the scale demonstrated excellent reliability, with an internal consistency coefficient α = 0.91 and an Omega coefficient of 0.93.

#### Psychological resilience

The Psychological Resilience was assessed using the Connor–Davidson Resilience Scale (CD-RISC), a validated measure of psychological resilience^37^, and the revised version was modified by Yu and Zhang^38^. The scale contains three dimensions of tenacity, strength and optimism, with good reliability and validity. There are 25 items in total. Using the Likert 5-level scoring method, higher scores indicate higher levels of psychological resilience. The Chinese version of the CD-RISC demonstrated a reliability coefficient of 0.91. Additionally, the internal consistency alpha values for the three factors were as follows: Factor 1 had an alpha value of 0.88, Factor 2 had an alpha value of 0.80, and Factor 3 had an alpha value of 0.60. In this study, the internal consistency coefficient was 0.97 and an omega coefficient of 0.95.

#### Need to belong scale

Need to Belong was measured by Leary^32^. It is used to measure individuals' need for acceptance and belonging (e. g. "If others refuse me, I will be bothered about it"). The scale has been widely employed in numerous studies conducted in China, demonstrating robust reliability and validity^39,40^. The scale is consisted by 10 questions with 5 rating, 1 as "totally inconformity" and 5 as "totally compliant". when the subject score was higher, you will get a stronger need to belong. The Cronbach'sαcoefficient in this study exceeded 0.80, while the omega consistency coefficient was found to be 0.78, indicating a high level of reliability for the study data.

### Statistical analyses

First, IBM SPSS29.0 statistical software was used for data analysis,including descriptive statistics and correlation analysis of Physical Exercise Behavior, Psychological Resilience, Social Sensitivity, Need to Belong and other variables. Also the common method bias was tested by the Harman uni-factorial test. Second, in this study, the mediation variables may form a mediation chain, and the predictor variables have indirect effects on the outcome variables through the mediation chain, model 6 in the macro program PROCESS of SPSS was used to conduct the mediating effect test^41^, it mainly tests the direct effect relationship between Physical Exercise Behavior and Psychological Resilience; The mediating effect of Social Sensitivity and Need to Belong; The Chain mediating effect of Physical Exercise Behavior and Psychological Resilience. Third, AMOS26.0 software was used to test the fitting degree of the mediating model between Physical Exercise Behavior and Psychological Resilience.

### Declarations

These study was reviewed and approved by the Ethics Committee of Institute of Psychology, School of Physical Education at Shandong University of Science and Technology of China, and all participants signed an informed consent form. All procedures were in accordance with the ethical standards of the responsible committee on human experimentation and with the Helsinki Declaration.

## Results

### Common method deviation test

In this study, all data were collected through a self-presentation questionnaire survey, which may introduce common method bias. To address this issue, three reverse questions were incorporated into the Social Sensitivity Scale during the questionnaire design process. Data collection was conducted through on-site filling, on-site answering, and on-site recovery procedures. Additionally, Harman's Single-factor Test was employed to perform exploratory factor analysis on all univariate unrotated items included in the scale. The analysis revealed the presence of seven factors with eigenvalues greater than 1, the explained variation for the maximum factor was 32.2%, which did not meet the recommended criterion of 40% as suggested by Hair et al.^42^ Consequently, no significant common methodological bias was observed in this study.

### Descriptive statistical and correlation analysis

As shown in Table 1, the correlation coefficients of physical exercise behavior, psychological resilience, social sensitivity and need to belong are all statistically significant. The correlation analysis shows that physical exercise behavior is positively correlated with psychological resilience and need to belong (*p* < 0.01, *P* < 0.05), also there was a significant negative correlation with social sensitivity (*p* < 0.01). Also, there are Sex differences in Physical Exercise Behavior (*P* < 0.01), Age has no correlation with each index. A chi-square test was further performed for Teenagers of different sex, As can be seen from Table 2, female had a higher Physical Exercise Behavior than males (*p* < 0.001), The Cohen's d value was utilized to quantify the magnitude of the sex difference, Cohen's d = 0.64, revealing a substantial disparity, and the difference was not significant for the other indicators.

**Table 1:** Descriptive statistics and correlation analysis.

	M	SD	Sex	Age	Physical exercise behavior	Psychological resilience	Social sensitivity	Need to belong
Sex	1.61	0.488	1					
Age	15.7	0.598	0.170	1				
Physical exercise behavior	2.65	0.856	− 0.301**	0.030	1			
Psychological resilience	3.65	0.653	− 0.026	0.047	0.194**	1		
Social sensitivity	3.18	0.694	0.008	0.031	− 0.085**	0.050*	1	
Need to belong	3.46	0.584	− 0.030	0.023	0.050*	0.470**	0.394**	1

N = 1106. *p < 0.05; **p < *0.01.*

**Table 2:** Differences in gender.

	Gender	Number	M ± SD	Chi-square	*p*
Physical exercise behavior	Female	433	2.97 ± 0.88	131.13	0.000***
	Male	673	2.45 ± 0.77		
Psychological resilience	Female	433	3.68 ± 0.72	93.05	0.05
	Male	673	3.64 ± 0.60		
Social sensitivity	Female	433	3.17 ± 0.72	57.32	0.25
	Male	673	3.19 ± 0.68		
Need to belong	Female	433	3.48 ± 0.67	49.73	0.08
	Male	673	3.44 ± 0.52		

N = 1106. ***p < *0.001.*

### Structural equation model construction and testing

The fit of the measured model and actual collected data was assessed using Amos26.0 software through rigorous testing procedures. After modification, the results indicated that the χ^2^/df ratio met the criterion of being less than 3. Additionally, RMESA demonstrated excellent adaptability within a range of 0.08. Furthermore, the CFI, AGFI, and GFI all surpassed the recommended standard of 0.80 set by experts with values exceeding 0.90 (Table 3). This indicates that the mediating model fits exceptionally well^43^. The multivariate normality test yielded *P* values > 0.05, indicating that the data were normally distributed in a multivariate sense. After standardizing the data, the variance inflation factor (VIF) was less than 5, suggesting an absence of multicollinearity. Homogeneity of error variances was tested and found to be indicative of independent errors^44^.

**Table 3:** Model fit index of the mediating role.

χ^2^/df	CFI	AGFI	GFI	NNFI	RMESA
2.86	0.92	0.90	0.93	0.91	0.07

### Significance test of mediation effect

The correlation analysis result met the statistical requirements for further testing the mediating effect of physical exercise behavior and psychological resilience. Consequently, the model 6 of SPSS macro program PROCESS was made to perform the mediation effect test. Table 4 shows that physical exercise behavior significantly positively predicts psychological resilience (β = 0.148, *p* < 0.001), and hypothesis 1 is established. Next, after incorporating social sensitivity and need to belong into the regression equation, physical exercise behavior can significantly negatively predict social sensitivity (β = − 0.069, *p* < 0.01), positively predicts need to belong (β = 0.058, *p* < 0.01), social sensitivity can significantly negatively predict need to belong (β = -0.337, *p* < 0.001) and negatively predict psychological resilience (β = − 0.132, *p* < 0.001), need to belong is significantly positively predicts psychological resilience (β = 0.580, *p* < 0.001). At this time, physical exercise behavior can still predict psychological resilience (β = 0.119, *p* < 0.001).

**Table 4:** Regression analysis of the relationship between variables.

Effect	Item	Effect	SE	t	Boot LLCI	Boot ULCI
Direct effect	Physical exercise behavior-psychological resilience	0.119	0.020	6.0***	0.080	0.158
indirect effect	Physical exercise behavior-social sensitivity	− 0.069	0.024	− 2.83**	− 0.117	− 0.021
	Physical exercise behavior-need to belong	0.058	0.019	3.05**	0.020	0.095
	Social sensitivity-need to belong	− 0.337	0.023	− 14.51***	− 0.383	− 0.291
	Social sensitivity-psychological resilience	− 0.132	0.027	− 4.975***	− 0.184	− 0.080
	Need to belong-psychological resilience	0.580	0.032	18.38***	0.518	0.641
Total effect	Physical exercise behavior-psychological resilience	0.148	0.023	6.57***	0.103	0.192

*BootLLCI* lower limit, *BootULCI* upper limit.

N = 1106. **p < 0.01, ***p < *0.001.*

**Figure 2:**
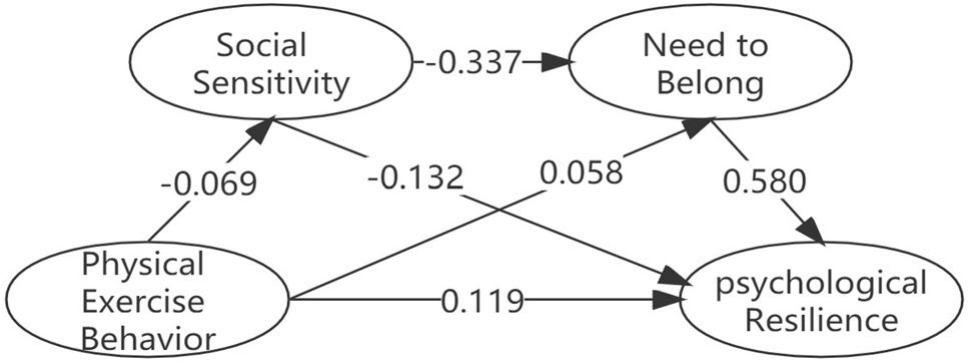
The chain-mediated mediation path of physical exercise behavior to psychological resilience.

The mediating effect size analysis results show that (see Table 5 and Fig. 2) social sensitivity and need to belong are used in physical exercise behavior and psychological resilience. There is a significant mediating effect between adaption, the indirect effect of the path with social sensitivity as the mediating variable is 0.009 (95%CI [0.002, 0.019]), the indirect effect of the path with need to belong as the mediating variable is 0.033 (95%CI [0.009,0.058]), the indirect effect of the path with psychological resilience social sensitivity and need to belong as the mediating variable is 0.014 (95%CI [0.003, 0.024]), the 95% confidence intervals of the three indirect paths do not contain the number 0, indicating that the three indirect effects have reached a significant level, and hypothesis 2, hypothesis3 and hypothesis 4 are valid.

**Table 5:** Mediating effect analysis of physical exercise behavior and psychological resilience.

Influence path	Indirect Effect	BootSE	95% confidence interval	
			BootLLCI	BootULCI
Physical exercise behavior-social sensitivity-psychological resilience	0.009**	0.004	0.002	0.019
Physical exercise behavior-need to belong-psychological resilience	0.033*	0.013	0.009	0.058
Physical exercise behavior-social sensitivity-need to belong-psychological resilience	0.014*	0.005	0.003	0.024

*BootLLCI* lower limit, *BootULCI* upper limit, *BootSE* standard error.

**p < *0.01*, *p < *0.05.*

## Discussion

### The relationship between physical exercise behavior and psychological resilience

This study demonstrates that physical exercise behavior significantly predicts the psychological resilience of adolescents, aligning with previous research^45,46^ and offering a novel perspective for advancing the understanding and promotion of the relationship between physical exercise behavior and psychological resilience in future studies. Psychological resilience, as an objective and universally observed phenomenon, enables individuals to effectively adapt and maintain a positive psychological state even in the face of trauma and frustration, thereby reflecting a constructive and robust psychological connotation. The positive impact of physical exercise behavior on adolescents is not only evident in their physical well-being but also in the promotion of their mental health. It has been demonstrated that they are able to recognize the advantages of self-improvement through engaging in physical activities, thereby maintaining a more frequent, positive, and consistent exercise routine^47,48^. Frequent engagement in physical exercise among students is associated with a more robust mental well-being, which can be attributed to the social support acquired through such activities. Communication and contact, cooperation and confrontation in sports facilitate the development of self-confidence and self-esteem among adolescents, as well as foster positive interpersonal relationships within the sporting context^15^. The psychological energy accumulated by physical exercise has improved the teenagers' ability to resist frustration and stress. When confronted with adversity and stress in life, the physiological stress response is mitigated while psychological resilience is bolstered^49,50^. Teenagers are in a critical period of cognitive and behavioral development, which coincides with a crucial phase for establishing healthy reserves. The cultivation of positive behavior and lifestyle choices serves as an essential determinant for mental well-being, while engaging in physical exercise fosters heightened sports experiences that contribute to enhanced psychological well-being. Therefore, it is imperative to enhance the level of physical activity among adolescents, improve their physical fitness, and foster their psychological well-being in order to enhance their social adaptability.

### The mediating role of social sensitivity

The present study reveals that social sensitization serves as a mediating factor in the relationship between physical exercise behavior and psychological resilience, highlighting its significance in comprehending this association. Engaging in physical exercise can facilitate adolescents to cultivate favorable and enduring interpersonal connections, fostering a confident and optimistic mindset. Consequently, the resulting sense of belonging and happiness diminishes social sensitivity, aligning with prior research findings^51,52^. Individuals with high social sensitivity pay more attention to the evaluation of peers or groups, thereby eliciting negative emotions such as reduced self-confidence. When faced with setbacks or difficulties, adolescents with high social sensitivity exhibit lower levels of psychological resilience compared to those with low social sensitivity. Consequently, these findings align with previous research results by indicating a negative association between social sensitivity and psychological resilience^24^. The level of social sensitivity plays a crucial role in the psychological adjustment of adolescents^53^. With the continuous advancement of social emotion and cognition, adolescents will increasingly prioritize and be vigilant towards others' attitudes, endeavoring to modify their behavior for acceptance, thereby heightening sensitivity to social evaluation; without proper guidance, this can lead to psychological issues. Notably, social experiences exhibit a more efficacious regulatory effect on highly sensitive adolescents^18,54^. Social experience exerts a more pronounced regulatory influence on highly sensitive adolescents^55^. As an intricately structured social endeavor, sports activities serve as a platform for communication and exchange, enabling teenagers to cultivate robust interpersonal relationships through interaction and cooperation. Whether it pertains to the organization and distribution in sports or the dynamics of cooperation and confrontation, these activities contribute to enhancing interpersonal communication and reducing social sensitivity. The positive experience facilitated by dopamine release during exercise proves advantageous for both physical and mental well-being among teenagers, effectively diminishing negative emotions such as sensitivity and inferiority. Moreover, a positive attitude can facilitate better adjustment of individuals' behavior and mood, mitigate social emotions such as low self-esteem and avoidance reactions in the face of setbacks, and contribute to higher levels of life satisfaction and subjective well-being^56^, thereby potentially enhancing psychological resilience.

### The mediating role of need to belong

This study further demonstrates that, in addition to social sensitivity, need to belong acts as a partial mediator between physical exercise behavior and psychological resilience, that is, physical exercise behavior can subsequently affect the psychological resilience of adolescents by influencing need to belong. The findings of this study are further corroborated by the existing body of research^29^. Participation in sports can enhance the sense of honor and collectivism, as adolescents prioritize interpersonal relationships during sporting activities, fostering a lasting attraction that cultivates a stable sense of belonging^57^. Simultaneously, whether it involves physical confrontation or skill acquisition, individuals face challenges in developing independently and require mutual support and assistance. The harmonious interpersonal environment experienced during these activities fulfills their strong need to belong, thereby reinforcing their engagement in physical exercise and contributing to mental well-being. By fostering a heightened sense of belongingness, adolescents can effectively confront adversity with a positive psychological state, ultimately cultivating robust psychological resilience^58,59^. The mechanism can employ the principles of self-determination theory, individual autonomy, and positive emotional experiences to motivate individuals to engage in actions that align with their interests and promote their own ability development. Once their physical and ability needs are met, a sense of belonging becomes an additional need. The cohesive force and close cooperation among teenagers in sports contribute to the positive development of their psychological health, thereby enhancing their psychological resilience^35^. The mission and responsibility of educators lie in promoting the physical and mental well-being of teenagers, ensuring they maintain a positive and optimistic mindset. This study suggests that engaging in physical exercise can enhance teenagers' psychological resilience by fulfilling their need to belong. Therefore, fostering a supportive social, sports, and cultural environment while encouraging physical activity participation can effectively cultivate positive psychological resources among teenagers and strengthen their overall resilience. Mental health also reciprocally influences physical exercise behavior, thereby fostering the advancement of both physical and mental well-being. Furthermore, the research findings additionally suggest that in order to enhance the psychological resilience of young individuals, interventions should not solely focus on external factors such as economic or policy-related aspects; rather, it is imperative to augment their awareness regarding physical activity, stimulate their motivation for exercise, and reinforce their need to belong within the context of engaging in physical exercise.

### The chain mediating effect of social sensitivity and need to belong

Through further analysis, this study reveals that the physical exercise behavior of adolescents can serve as a predictive indicator for psychological resilience, operating through the mediating factors of social sensitivity and need to belong. Teenagers develop a positive attitude and a strong sense of collective honor through engaging in physical exercise. The optimistic and confident mindset resulting from positive emotional experiences and an optimistic psychological state effectively diminishes their social sensitivity while enhancing their sense of belonging^51,52^. A higher need to belong can facilitate teenagers' emotional and mood regulation, leading to recognition and acceptance from the collective or others, thereby promoting psychological well-being. In the face of failure experiences, individuals exhibit reduced susceptibility to emotional fluctuations and demonstrate rapid recovery from negative emotions^24^. Given that socially sensitive children develop self-perception of social competence and interpersonal relationships during middle childhood^60,61^, the stage of middle school becomes a critical period for their physical and psychological development. Therefore, it is imperative for schools and families to effectively engage in educational efforts during this stage by promoting active participation in sports activities, imparting health-related knowledge, fostering healthy habits, enhancing mental well-being, and ultimately improving their ability to adapt to society.

Therefore, in accordance with the self-determination theory, engagement in physical exercise can foster adolescents' mental well-being and enhance their psychological resilience, while a significant inverse association exists between social sensitivity and the need to belong^48^. This study demonstrates the feasibility of chain mediation, indicating that social sensitive-need to belong can partially mediate the relationship between physical exercise behavior and teenagers' psychological resilience. The constructed mediation effect model partially elucidates the underlying mechanism of physical exercise behavior among adolescents in bolstering their psychological resilience, thereby offering valuable guidance for enhancing teenagers' psychological resilience, fostering a healthy mindset, and promoting social adaptability. Simultaneously, considering that teenagers primarily engage in activities within their families, communities, and schools, it is imperative to also consider environmental factors influencing their psychological resilience and physical exercise behavior^62^. The family environment exerts a continuous influence on exercise behavior tendencies and psychological resilience, while the community serves as an extension of this environment. As the closest setting to teenagers, its impact on their physical and mental well-being should not be overlooked^63^. The school environment serves as an inexhaustible catalyst and reservoir of strength for enhancing the well-being of adolescents, exerting a direct influence on promoting physical activity engagement and bolstering psychological resilience.

### Limitations and prospectives

This study investigates the association between physical exercise behavior and psychological resilience in teenagers, constructs a chain mediation model to elucidate the underlying mechanism of how physical exercise behavior influences adolescent psychological resilience, and holds significant theoretical and practical implications for comprehending the factors influencing adolescent psychological resilience. However, it is important to note that this study adopts a cross-sectional design and solely considers mediation variables without accounting for their causal effects; thus, establishing a definitive causal relationship remains beyond its scope, which should be addressed in future research. Simultaneously, future studies should also consider environmental factors that influence the psychological resilience and physical exercise behavior of adolescents. Additionally, it is worth exploring alternative data collection methods such as multi-mode and multi-angle approaches to mitigate potential reaction bias associated with the use of self-report scales in this study. Lastly, given the limitations imposed by research conditions, it is important to acknowledge that our sample only includes freshmen from a specific high school rather than encompassing all youth groups; this aspect should be taken into account in future investigations.

## Conclusion

Based on the self-determination theory, we propose a chain mediation model, specially, physical exercise behavior can significantly positively predict psychological resilience, among which, social sensitivity and need to belong play a significant mediating role between Physical exercise behavior and psychological resilience. In addition, the adoption of good physical exercise behavior can enhance the psychological resilience of adolescents by diminishing social sensitivity and augmenting the need to belong (Supplementary file).

### Supplementary Information

Supplementary Information.

## Data Availability

The original contributions presented in the study are included in the article/supplementary material, further inquiries can be directed to the corresponding authors.
